# Analytical findings in a non-fatal intoxication with the synthetic cannabinoid 5F-ADB (5F-MDMB-PINACA): a case report

**DOI:** 10.1007/s00414-021-02717-6

**Published:** 2021-12-18

**Authors:** Franziska Gaunitz, Hilke Andresen-Streichert

**Affiliations:** Institute of Legal Medicine, University of Cologne, Faculty of Medicine and University Hospital, Cologne, Germany

**Keywords:** New psychoactive substances, LC-ESI-MS/MS, Popeye 2G weed, *Spice*, Cardiac side effects

## Abstract

The case report centres on analytical findings from a *spice* sample (mixed with tobacco (as a *cigarette*) for consumption), and its corresponding plasma sample, smoked by a 31-year-old man who was attended by emergency services following collapse. The man was fully conscious and cooperative during initial medical treatment. Suddenly, he suffered a complete loss of self-control, whereupon the police was notified. The man encountered the police officers when exiting the apartment, at which point he threatened them with clenched fists and reached for a plant bucket in order to strike out in the direction of the officers. At the trial, he described himself as confused and as being completely overwhelmed, having lost self-control, suffered a panic attack and “just wanted to get out the situation”. Furthermore, he stated that he had no recollection of the incident. He feared death due to palpitations, heart pain, dizziness and repetitive anxiety states. Routine systematic as well as extended toxicological analysis of the plasma sample, taken approximately 2 h after the incident, confirmed the use of cannabis and *spice*. Plasma concentrations of THC, OH-THC and THC-COOH were 8.0 μg/L, 4.0 μg/L and 147 μg/L, respectively. Furthermore, analysis confirmed uptake of 5F-ADB (5F-MDMB-PINACA) via detection of both 5F-ADB and the 5F-ADB *N*-(5-OH-pentyl) metabolite. The *spice* sample additionally contained 5F-MDMB-PICA, which was not detected in the plasma sample. A differentiation between a possible co-use and a recent use of cannabis was not possible. In summary, this case once more underlines the health risks of *spice* use.

## Introduction

According to the European Drug Report 2019, published by the European Monitoring Centre for Drugs and Drug Addiction (EMCDDA), synthetic cannabinoids—also known as *herbal mixtures* or *spice*—represent an important group among the “New Psychoactive Substances” (NPS) [1]. In total, more than 730 NPS are being monitored by the EMCDDA (as of June 6th 2019) [1]. In Germany, as in many countries in Europe and worldwide, NPS are prohibited substances. NPS are also registered on the World Anti-Doping Agency’s (WADA) Prohibited List. Several NPS are subject to the German Narcotics Act (BtMG), with the remainder being subject to the “NPS law” (NpSG). The marketing of synthetic cannabinoids as “legal alternatives to cannabis”, “natural herbs”, “harmless” or “safe herbal mixtures” may contribute for their popularity [2]. Most users of synthetic cannabinoids are described in the literature as cannabis users who are curious about the effects of smoking “legal alternatives to cannabis” [2–5]. *Spice* is usually used in combination with tobacco, or with cannabis, as “cigarettes” or “joints” [6].

Synthetic cannabinoids as well as cannabis’ main active substance tetrahydrocannabinol (THC) both interact with the cannabinoid receptors CB_1_ and CB_2_, whereby THC acts as a partial agonist and most of the synthetic cannabinoids act as full agonists [7, 8]. Due to their effect as a full agonist and due to their higher affinity to CB_1_ and/or their higher potency at CB_1_ compared to THC [9–13], significantly stronger (side) effects, and thus significantly increased toxicity, must be assumed. To date, a number of hospital cases have been reported with severe to lethal intoxications following the use of synthetic cannabinoids [14–53]. Side effects of synthetic cannabinoid use regarding the central nervous system include anxiety, agitation, panic attacks, irritability, aggression, changes in mood and perception, confusion, paranoia, psychosis, seizures and coma. Cardiovascular side effects are tachycardia, arrhythmia, and heart and chest pain, respectively. Among the potentially lethal effects, circulatory collapse, respiratory depression and cardiac arrest are listed in particular. [2, 9, 37, 38, 45, 54–56]

The case presented here describes an incident in September 2018 involving the synthetic cannabinoid 5F-ADB (also known as 5F-MDMB-PINACA). According to the EMCDDA Europol joint report, 5F-ADB was first detected on the European drug market in September 2014 [57]. In 2015, this synthetic cannabinoid was reported to the UNODC (United Nations Office on Drugs and Crime) early warning system both by Hungary and Japan in 2015 [11], involving “a nationwide outbreak” with it being associated with, e.g., several motor vehicle collision cases in Japan in the summer of 2014 [58]. In 2017, the EMCDDA and Europol classified herbal material (*spice*) as the most popular form of 5F-ADB seizures, in comparison to other forms such as (white) powder, liquids (to be used in e-cigarette devices) and blotters [57]. Therefore, the primary route of administration would most likely be via inhalation, either by smoking *spice* as a “cigarette” or “joint”, or utilizing a vaporizer [59].

In Germany, 5F-ADB was one of the most popular synthetic cannabinoids in 2016 until it became subject to the BtMG in July 2016. In China, where the originators of synthesis and distribution of synthetic cannabinoids are presumed to be, 5F-ADB, among others, was banned by law in 2018 [60].

**Fig. 1:**
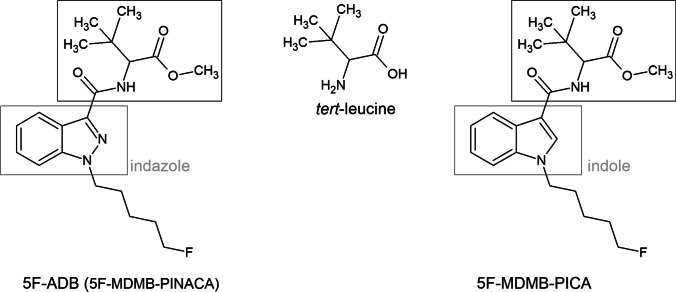
Structure of 5F-ADB (on the left) and 5F-MDMB-PICA (on the right) with an indazole or indole core, respectively, a fluorinated pentyl side chain, carboxamido linker and *tert*-leucine methyl ester bridge rest. Both synthetic cannabinoids were detected in the here presented *spice* sample

5F-ADB belongs to a comparably newer series of synthetic cannabinoids featuring amino acid derivatives, e.g. *tert*-leucine or valine. 5F-ADB is an indazole-based synthetic cannabinoid from the indazole-3-carboxamide family, thus possessing a carboxamido linker. The structure of 5F-ADB is further characterized by a fluorinated pentyl side chain and a *tert*-leucine methyl ester bridge rest (see Fig. 1).

According to Banister et al., 5F-ADB demonstrates subnanomolar half-maximal effective concentration (EC_50_ = 0.59 nM), which was shown to be about 30 times lower than the EC_50_ value of JWH-018 (EC_50_ = 18 nM) and about 290 times lower than the EC_50_ value of THC (EC_50_ = 171 nM), emphasizing the extremely high potency of 5F-ADB [11]. This was also demonstrated by Antonides et al., who determined an about 25 timer lower EC_50_ value for the *S*-enantiomer of 5F-ADB (EC_50_ = 1.78 nM) than for JWH-018 (EC_50_ = 45.1 nM) [61]. They also showed significant differences in EC_50_ values between the *S*-enantiomer of 5F-ADB and the corresponding *R*-enantiomer, with the latter having an EC_50_ value of 131 nM [61].

5F-ADB was shown to be an agonist and partial activator of CB_1_ [12]. Furthermore, Asaoka et al. observed 5F-ADB to significantly increase the spontaneous firing rate of dopaminergic neurons ex vivo, whereby 5F-ADB did not affect the spontaneous firing rate of serotonergic neurons, suggesting 5F-ADB activation of local CB_1_ and stimulating midbrain dopaminergic systems [62].

5F-MDMB-PICA, which plays a minor role in this particular case as it was only detected in the *spice* sample, and not in the plasma sample, is structurally related to 5F-ADB. Compared to 5F-ADB, 5F-MDMB-PICA contains an indole core instead of an indazole core (see Fig. 1), and 5F-MDMB-PICA is slightly more potent compared to 5F-ADB (EC_50_ = 0.45 nM) [11]. At the time of the event, 5F-MDMB-PICA was not subject to the BtMG, although it was subject to the NpSG. 5F-MDMB-PICA became subject to the BtMG in July 2020.

The use of 5F-ADB has been associated with acute intoxications [22, 63] and with a number of fatal mono-intoxications [29, 64] as well as poly-intoxications (co-use of the synthetic stimulant diphenidine [53], MAB-CHMINACA [65], or different substances—including synthetic cannabinoids [14, 63, 66]). Regarding the reported cases of acute intoxications, adverse effects were severe and included the following: altered or loss of consciousness [22, 58, 63]; severe headache, dizziness, confusion, anxiety, psychosis, (psychomotor) agitation, mydriasis and temporary amnesia [22]; loss of memory and tachycardia [58]; changing moods and physical collapse [63]. The EMCDDA Europol joint report of 2017 listed 35 acute intoxications reported in 2016 (in Hungary and the UK) and 24 fatal intoxications reported between 2015 and 2017 (in Germany and the UK) [57].

Halter et al. [67] investigated the prevalence of 5F-ADB and 5F-MDMB-PICA in Germany between January 2016 and September 2019 in quarterly (Q1–Q4) analyses of a total of 987 *spice* samples, purchased from German-language online shops, 4291 serum samples and 24369 urine samples. During Q1 and Q2 of 2016, the proportion of *spice* samples containing 5F-ADB was 30%, falling to 5% in Q3 and to 0% in Q4 of 2016. However, in their opinion, the authors observed an “unexpected” increase from Q1 of 2017 (16%; unpublished percentage data, received on request from the authors) to Q3 of 2018 (40%), followed by another decrease from Q4 of 2018 (26%) to Q3 of 2019 (0%). This was also reflected by the analysis of the biological samples. Interestingly, first detection of 5F-MDMB-PICA occurred with the decrease of 5F-ADB proportion in Q3 of 2016 and disappeared with the increase of 5F-ADB in 2017. In 2018, both 5F-ADB and 5F-MDMB-PICA were detected in *spice* samples, whereby the proportion of 5F-ADB was approximately 40% and of 5F-MDMB-PICA about 5% in Q3. As a conclusion, Halter et al. suggested that BtMG and NpSG had only a limited effect on the appearance of 5F-ADB and 5F-MDMB-PICA in *spice* samples.

**Fig. 2:**
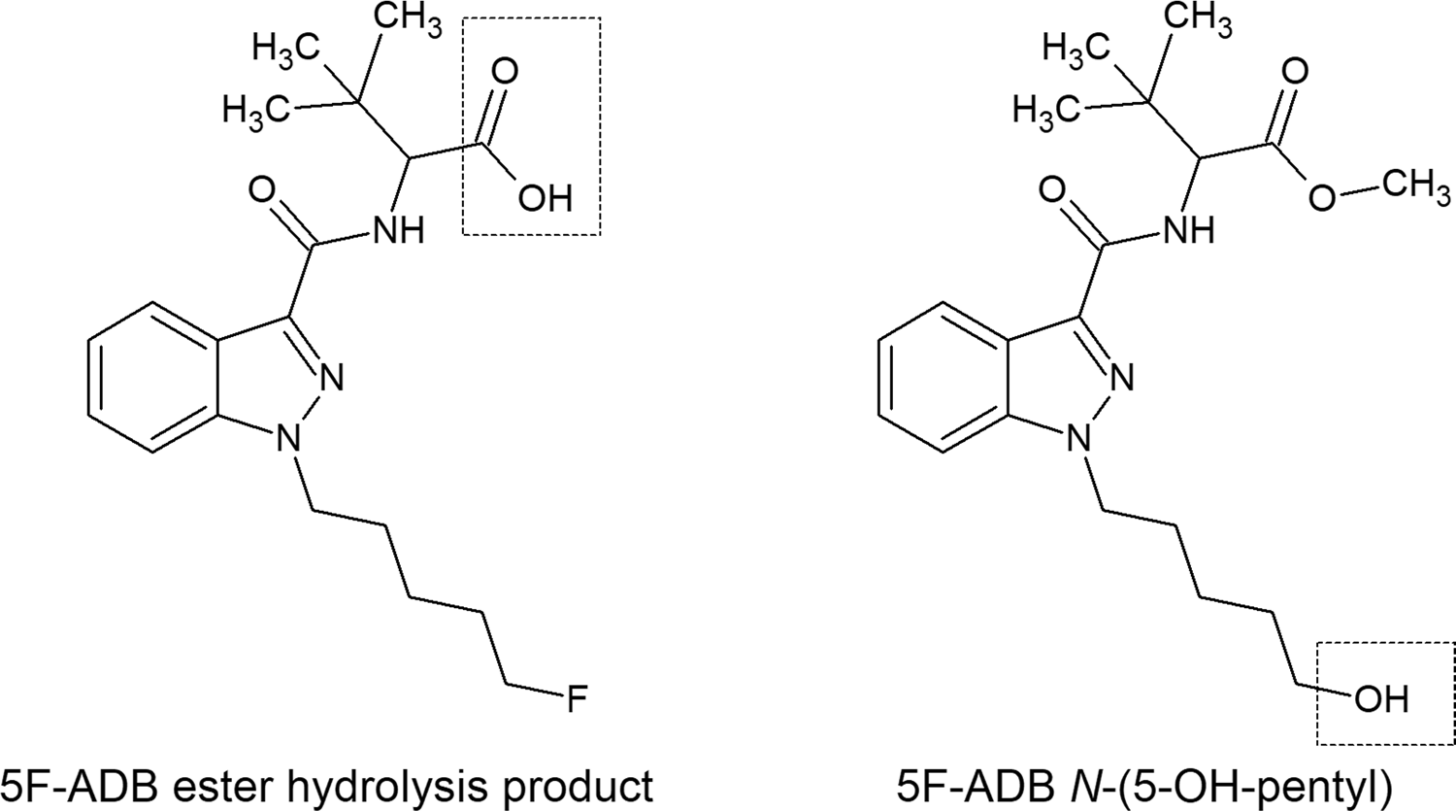
Structures of considered 5F-ADB metabolites in this case: the 5F-ADB ester hydrolysis product (“metabolite M2”, on the left) and the 5F-ADB N-(5-OH-pentyl) (“metabolite M7”, on the right)

5F-ADB undergoes extensive hepatic metabolism. Results of metabolism studies with pooled human liver microsomes (pHLM) as well as results of both blood and urine screenings have been previously published [22, 53, 63, 68, 69]. According to the literature, the metabolism of 5F-ADB is dominated by ester hydrolysis, defluorination (revealing the *N*-(5-OH-pentyl) metabolite) and monohydroxylations. In the here presented case, metabolite screening was limited to the 5F-ADB ester hydrolysis product and 5F-ADB *N*-(5-OH-pentyl) (see Fig. 2).

## Case history

Following the use of cannabis, a 31-year-old man with drug experience smoked *spice* for the first time, mixed with tobacco (as a cigarette). He bought this *spice* sample online from an internet shop, as it was freely and “legally” available. However, police officers only managed to locate the remainder of a “joint” at the scene.

He smoked a *spice *cigarette while alone in his sister’s apartment. When she returned home from work at 3.30 pm, her brother’s manner and behaviour were “normal”. He left to take a shower, when suddenly (at about 4.15 pm) his sister heard him screaming loudly, shouting “My heart, my heart!” after which he collapsed.

His sister immediately called the emergency services. She described him as very restless, unable to stand up, pale and tremulous. During treatment by two paramedics, the man was fully conscious and responsive, calm and cooperative, but a little stimulated and nervous in his behaviour. However, he then suddenly stood up, hugged his sister, told her “I love you” after which he became increasingly aggressive and threatening towards those present. From this moment on, the man exhibited erratic and nervous behaviour. He jumped up from his chair and pulled his sister’s hair. One of the paramedics had to throw himself to the floor to evade a physical attack. He pushed the paramedics away, was heard (by other residents of the apartment house) to continue to yell loudly and he then attempted to exit the apartment. During this disturbance, the police had been called to support the paramedics.

The man finally exited the apartment and rushed out into the corridor, where he encountered the police officers and began threatening one of the police officers with his clenched fists. He also attempted to beat and kick them. The man then reached for one of the plant buckets located there and tried striking out in the direction of one police officer, who was able to avoid the attack. He discarded the plant bucket and pursued the police officer, after which he threatened a second police officer in a similar manner. After a second miss, the man run away from the scene. His sister was finally able to calm him down and bring him back to her apartment. In the interim, police had called for reinforcements in order to take the intoxicated man into custody. The arrest proceeded without resistance. The entire incident lasted at least 15 min.

The man was finally taken to the hospital, where blood samples were obtained at 6.00 pm, approximately 2 h after the incident. According to the medical report, his pupils were slightly dilated. The drugs’ influence on his external appearance was only slightly noticeable. He appeared to be minimally dazed. An alcohol breath test was negative. A drug pre-test was not conducted. He was handed over to the custody of his sister later that evening. Since a charge of assault on police officers was submitted, the case went to court.

In court, the man stated that he was “no longer himself” after smoking *spice*. He described himself as confused, as being completely overwhelmed, having no perception of his surroundings—having lost control of his senses—and stated that he just wanted to get away and get out of the situation. He could not recall the incident. He had thought he was dying. He remembered suffering from palpitations, heart pain, dizziness and repetitive anxiety states. The description of his behaviour, along with results from systematic toxicological analysis (STA), prompted a judgement of both situation-inadequate behaviour and a substance-dependant decrease in controllability.

## Materials and methods

### Samples

**Fig. 3:**
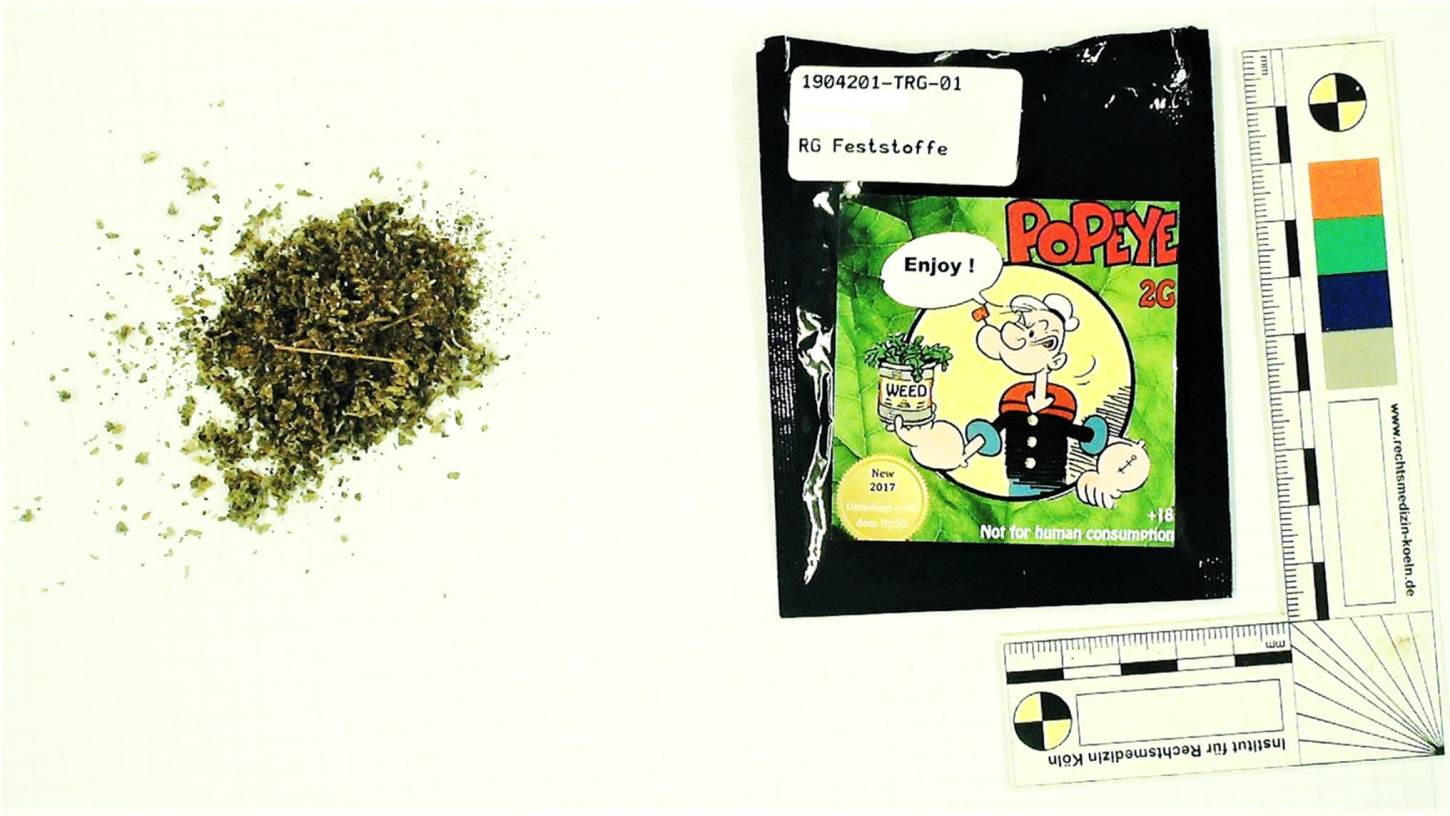
Label of “Popeye 2G, weed” (downloaded from https://lsd-blotter.com/legal-Smoke/Popeye-Legal-2g-R%C3%A4uchermischung, on December 18th 2020) (on the left) and *spice* sample of the here presented case (on the right)

The samples available for STA were 6 mL potassium fluoride (NaF)–stabilized blood sample (and a corresponding plasma sample) and a 1 g *spice* sample. The *spice* sample was green plant material, packed in a small bag with a clamp closure and labelled with “Popeye 2G, weed” (see Fig. 3). A blood sample without any additives and a serum sample were not available, respectively.

### Chemicals

#### Chemicals and reagents used for immunochemical examinations

Testing for the presence of amphetamine and amphetamine derivatives (AMP), benzodiazepines (BZ), cocaine and cocaine metabolites (COC), methadone and EDDP (ME), opiates (OP) and cannabinoids (CAN) was carried out using inhomogeneous enzymatic immunoassay kits (Immunalysis® Microplate EIA Kits, Abbott Rapid Diagnostics Germany GmbH, Cologne, Germany). *Pro analysi* ethylene diamine tetra-acetic acid (EDTA) disodium salt di-hydrate was purchased from VWR (Langenfeld, Germany).

#### Chemicals and reagents used for “general unknown” screening

*Ortho*-phosphoric acid (H_3_PO_4_; 85%) was purchased from Carl Roth (Karlsruhe, Germany). Both *HPLC gradient grade* acetonitrile (ACN) and water were also obtained from Carl Roth (Karlsruhe, Germany). *Pro analysi* disodium hydrogen phosphate (Na_2_HPO_4_; anhydrous), potassium dihydrogen phosphate (KH_2_PO_4_; anhydrous), sodium hydroxide (NaOH) as well as potassium hydroxide (KOH) pellets and 1-chlorbutane were obtained from Merck (Darmstadt, Germany). Hexobarbital, ethyl-nordiazepam and 2-methyl-1-phenyl-2-propyl-hydroperoxide (MPPH) were obtained from Lipomed GmbH (Weil am Rhein, Germany).

#### Chemicals and reagents used for GC-EI-MS/MS analysis of cannabinoids

*HPLC gradient grade* methanol (MeOH) was purchased from Carl Roth (Karlsruhe, Germany). *HPLC gradient grade* ACN was obtained from Th. Geyer (Lohmar, Germany). *Pro analysi* acetic acid was obtained from Merck (Darmstadt, Germany). N-methyl-N-(trimethylsilyl) trifluoroacetamide (MSTFA) was obtained from Macherey Nagel (Düren, Germany). Certified reference materials of ∆9-tetrahydrocannabinol (THC) and its metabolites 11-hydroxy-THC (OH-THC) and 11-nor-carboxy-THC (THC-COOH) as well as of its deuterated forms (THC-d3, OH-THC-d3 and THC-COOH-d3) were purchased from LGC standards GmbH (Luckenwalde, Germany).

#### Chemicals and reagents used for LC-ESI-MS/MS analysis

*LC-MS gradient grade* water and ACN were purchased from Carl Roth (Karlsruhe, Germany). *LC-MS gradient grade* ammonium formate (NH_4_COO) and formic acid (HCOOH) were obtained from Merck (Darmstadt, Germany). LC solvent consisted of 2 mM NH_4_COO buffer with 0.1% HCOOH (solvent A) and ACN with 0.1% HCOOH (solvent B). LC solvent for dilution of the *spice* sample consisted of 60% A and 40% B (according to the initial conditions of the LC gradient). Analytical reference material of 5F-ADB (5F-MDMB-PINACA), 5F-NPB-22 MDMB-CHMICA and 5F-MDMB-PICA, as well as analytical reference material of 5F-ADB metabolites 5F-ADB *N*-(5-OH-pentyl) (“metabolite M2”) and 5F-ADB ester hydrolysis product (“metabolite M7”), was obtained from Cayman Chemical (Ann Arbor, MI, USA) via LGC standards GmbH (Luckenwalde, Germany).

### Preparation of the spice sample

Two aliquots of 25 mg were weighed in two 5-mL volumetric flasks and extracted with ACN, utilizing an ultrasonic bath. For further analysis, the 25 mg/5 mL samples were diluted with ACN (1:100 and 1:1000).

### Routine systematic toxicological analysis

Routine STA of the plasma sample consisted of immunochemical examinations, confirmation analysis performed by means of gas chromatography coupled to tandem-mass spectrometry with electron ionization (GC-EI-MS/MS) and a “general unknown” screening by means of high-performance liquid chromatography coupled with diode-array detection (HPLC-DAD).

### Immunochemical examinations

Prior to the immunochemical examinations, a 400-μL aliquot of the plasma sample was mixed with an equal volume of 0.1 M EDTA solution. Immunchemical examinations were conducted on a TECAN Freedom EVOlyzer® 100:2 system (TECAN Group Ltd., Maennedorf, Switzerland). Evaluation of the immunochemical examination results was based on validated cut-off values.

### “General unknown” HPLC-DAD screening

HPLC-DAD screening was only performed on the plasma sample. The methodology has been comprehensively described elsewhere [70]. In a divergence from this method description, sample preparation only consisted of an alkaline liquid-liquid extraction (LLE) with 1-chlorbutane. Extraction process was supported using a Heidolph Multi Reax shaker (Heidolph Instruments GmbH, Schwabach, Germany) at approximately 1500 rpm. The internal standard (ISTD) solution contained not only hexobarbital (30 mg/L HPLC mobile phase) and ethyl-nordiazepam (2 mg/L HPLC mobile phase), but also MPPH (10 mg/L HPLC mobile phase). The evaporated extract was reconstituted with 50 μL HPLC mobile phase.

### GC-EI-MS/MS analysis of cannabinoids

The in-house method used for the analysis of cannabinoids in plasma covers the analysis of THC and its metabolites OH-THC and THC-COOH. Analytes are derivatised with MSTFA and extracted via automatic solid-phase extraction. ISTD consists of THC-d_3_, OH-THC-d_3_ and THC-COOH-d_3_. The stationary phase was a Zebron ZB-5MSi (30 m × 0.25 mm × 0.25 μm) from Phenomenex (Aschaffenburg, Germany). Details of this methodology are described elsewhere [71].

### LC-ESI-MS/MS target screening for synthetic cannabinoids

Target screening for synthetic cannabinoids was performed by means of liquid chromatography coupled to tandem-mass spectrometry with electrospray ionization (LC-ESI-MS/MS). The LC-ESI-MS/MS screening for synthetic cannabinoids was performed on the plasma sample and the *spice* sample. Furthermore, a drug-free plasma sample was spiked with synthetic cannabinoids for retention time and multiple reaction monitoring (MRM) ratio adjustment. Prior to the screening, the plasma samples were extracted via alkaline LLE, in the same fashion to this used for the “general unknown” HPLC-DAD screening. However, contrary to this, the evaporated extract was reconstituted in 50 μL LC mobile phase. The LC-ESI-MS/MS instrumentation and the LC method have already been described elsewhere [72]. Regarding the published “standard” LC gradient, initial conditions were 40% of solvent B, held for 1.0 min, increased to 90% over 6.0 min and to 98% over 0.5 min, kept at 98% for 2.0 min and finally returned to 40%. LC stop-time was 10.0 min and post runtime 2.3 min, resulting in a total runtime of 12.3 min. Regarding the optimized LC gradient in order to differentiate between 5F-ADB (5F-MDMB-PINACA) and 5F-NPB-22, initial conditions were maintained, held for 2.0 min, increased to 50% of solvent B 2 min and held for 2.5 min, further increased to 98% within 1 min and kept at 98% for 2.0 min and finally returned to 40%. While ESI parameters were retained, MS/MS parameters differ from [72] with regard to MRM transitions (see Table 1).

**Table 1:** Synthetic cannabinoids tested positive in the first target screening: abbreviations, molecular formulas, ion transitions, fragmentor voltages, collision energies (CE) and retention times (t_R_)

Synthetic cannabinoid	Molecular formula	Precursor ion [m/z] (nominal mass)	Product ions [m/z] (nominal masses)	Fragmentor voltage [V]	CE [V]	t_R_ [min] (“standard” LC gradient)	t_R_ [min] (optimized LC gradient)
5F-ADB (5F-MDMB-PINACA)	C_20_H_28_FN_3_O_3_	378	*233* 145	110	*21* 45	4.2	4.7
5F-NPB-22	C_22_H_20_FN_3_O_2_	378	*233* 145	110	*13* 41	4.2	4.5
5F-MDMB-PICA	C_21_H_29_FN_2_O_3_	377	*232* 145	110	*13* 45	3.7	–

In addition to the target screening for synthetic cannabinoids, a further target screening for 5F-ADB metabolites was conducted. The measurements were limited to a qualitative analysis for 5F-ADB *N*-(5-OH-pentyl) (“metabolite M2”) and the 5F-ADB ester hydrolysis product (“metabolite M7”), which were available for purchase (see Table 2).

**Table 2:** Analysed/considered 5F-ADB metabolites: abbreviations, molecular formulas, ion transitions, fragmentor voltages, collision energies (CE) and retention times (t_R_)

5F-ADB metabolite	Molecular formula	Precursor ion [m/z] (nominal mass)	Product ions [m/z]: (nominal masses)	Fragmentor voltage [V]	CE [V]	t_R_ [min] (LC gradient screening method)
5F-ADB *N*-(5-OH-pentyl) (“metabolite M2”)	C_20_H_29_N_3_O_4_	376	*213* 145	110	*25* 45	1.6
5F-ADB ester hydrolysis product (“metabolite M7”)	C_19_H_26_FN_3_O_3_	364	*233* 145	110	*25* 45	2.1

## Results and discussion

### Routine systematic toxicological analysis

By means of immunochemical examinations, AMP, BZ, COC, ME and OP were found to be negative in the plasma sample as the detected concentrations were below the validated threshold values. The positive immunochemical result for CAN was verified by the positive findings of THC, OH-THC and THC-COOH in the plasma sample by means of GC-EI-MS/MS analysis (see Table 3). As a plasma sample and no serum sample was measured, slightly lower cannabinoid concentrations could be assumed for further evaluation [73]. However, the THC concentration detected proved an effect at the time of the blood sampling as well as at the time of the incident. Due to the THC-COOH concentration, it was assumed that the man is likely to be a regular cannabis user [74, 75]. In cases of regular cannabis use, a co-detection of THC and OH-THC does not necessarily prove consumption in a close time interval prior blood sampling.

**Table 3:** Results of the systematic and advanced toxicological analysis on the plasma sample as well as of the *spice* sample (*n. t.* not targeted)

	Plasma concentration [μg/L]	*spice* sample
GC-EI-MS/MS analysis of cannabinoids		
THC	8.0	n. t.
THC-OH	4.0	n. t.
THC-COOH	147	n. t.
HPLC-DAD screening		
4-MAA (metamizole metabolite)	~ 8700	n. t.
Caffeine	positive	n. t.
LC-ESI-MS/MS target analysis of synthetic cannabinoids		
5F-ADB (5F-MDMB-PINACA)	positive	positive
5F-ADB *N*-(5-OH-pentyl) (“metabolite M2”)	positive	n. t.
5F-ADB ester hydrolysis product (“metabolite M7”)	negative	n. t.
5F-MDMB-PICA	negative	positive

THC use usually results in sedation, euphoria and temporal distortion. As THC acts as a partial agonist at the cannabinoid receptors CB_1_ and CB_2_, it is prone to share side effects with synthetic cannabinoids, such as confusion, paranoia and psychosis, and cardiovascular side effects (see “Introduction”).

HPLC-DAD screening revealed evidence of *N*-methyl-4-aminoantipyrine (4-MAA) and caffeine (see Table 3). The detection of the pharmacologically active metabolite 4-MAA proved an uptake of metamizole (dipyrone), which is both an analgesic, antispasmodic, antipyretic and antiphlogistic/anti-inflammatory substance [76]. It is used for indications such as colic pain or headache [76]. Concentration of 4-MAA was below the therapeutic range (the minimal effective concentration is approximately 10,000 μg/L plasma [77]). However, taking into account the time between incident and blood sampling, an effect of metamizole or 4-MAA, respectively, at the time of the incident is to be assumed. According to the literature, intake of metamizole can cause cardiovascular side effects (i.e. hypertension and arrhythmia) [76].

The positive finding for caffeine is likely explainable by drinking of caffeine-containing beverages (e.g. coffee, green or black tea) as this plant-based alkaloid naturally occurs in coffee beans and tea leaves. An uptake of caffeine-enriched (“energy”) drinks or caffeine-containing analgesics is also possible. Within this case, the finding has no toxicological relevance.

### LC-ESI-MS/MS target screening for synthetic cannabinoids

**Fig. 4:**
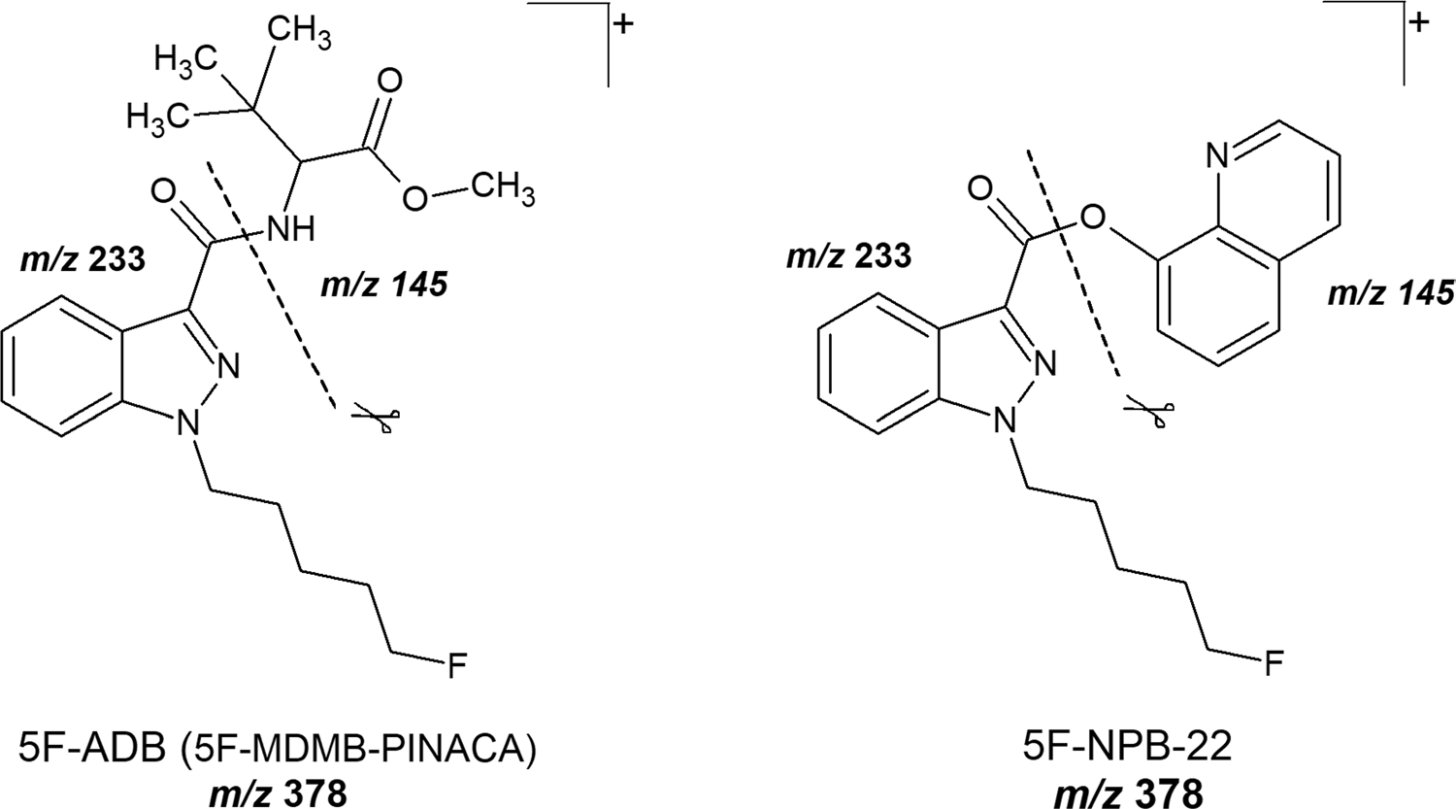
Fragmentation of 5F-ADB (5F-MDMB-PINACA) and 5F-NPB-22 during ESI (+) collision-induced dissociation (CID)

**Fig. 5:**
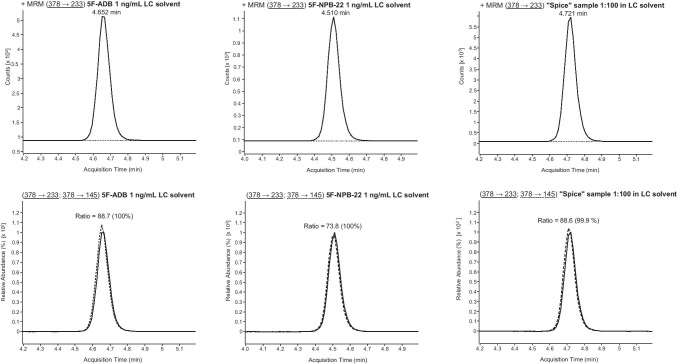
Differentiation between 5F-ADB (5F-MDMB-PINACA) and 5F-NPB-22 via LC-ESI-MS/MS analysis (using the optimized LC gradient) of the *spice* sample (1:100 solution). MRM transitions and ion ratios of 5F-ADB (5F-MDMB-PINACA), 5F-NPB-22 standard solutions (1 ng/mL LC solvent each) and *spice* sample (1:100 solution in LC solvent). For explanation: the respective sample can be found in the columns, the upper row shows the first MRM transition and the lower row the second MRM transition as well as the ion ratios

Initially, LC-ESI-MS/MS target screening for synthetic cannabinoids in the plasma sample revealed a positive result for 5F-ADB (5F-MDMB-PINACA) and 5F-NPB-22. These synthetic cannabinoids were also detected in the *spice* sample. However, as 5F-ADB (5F-MDMB-PINACA) and 5F-NPB-22 possess similar MRM transitions (see Fig. 4) and retention times (see Table 1), the LC gradient of this method had to be optimized in order to enable a differentiation between 5F-ADB (5F-MDMB-PINACA) and 5F-NPB-22. Following confirmation analysis and performance of the optimized LC gradient to the samples, the presence of 5F-NPB-22 was excluded (see Fig. 5). Due to the positive finding of 5F-ADB (5F-MDMB-PINACA) (see Fig. 7), the plasma sample was additionally screened for its metabolites. Examinations revealed positive results for 5F-ADB *N*-(5-OH-pentyl) (“metabolite M2”) (see Fig. 8). The 5F-ADB ester hydrolysis product (“metabolite M7”) was not detectable in the plasma sample.

**Fig. 6:**
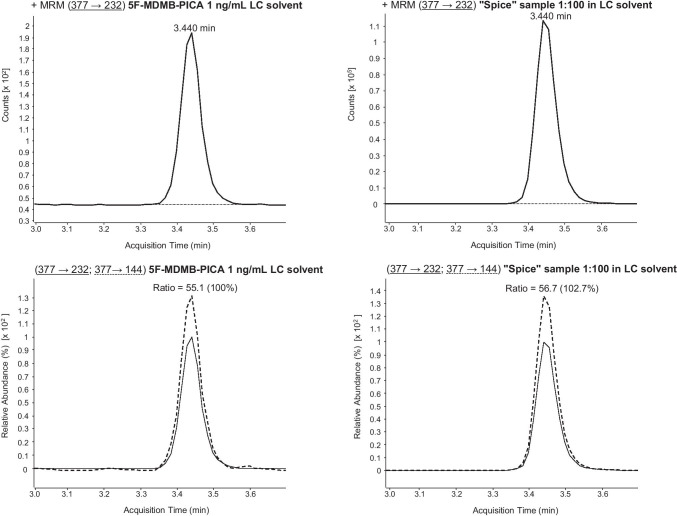
5F-MDMB-PICA confirmation via LC-ESI-MS/MS (using the standard LC gradient) analysis of the *spice* sample (1:100 solution). MRM transitions and ratios of 5F-MDMB-PICA standard solution (1 ng/mL LC solvent) and *spice* sample (1:100 solution in LC solvent). For explanation: the respective sample can be found in the columns, the upper row shows the first MRM transition and the lower row the second MRM transition as well as the ion ratios

Regarding the *spice* sample, there was additional evidence of the synthetic cannabinoid 5F-MDMB-PICA (see Fig. 6). However, as this synthetic cannabinoid was found to be negative in the plasma sample, further examinations were waived. All results are summarized in Table 3.

## Conclusions

**Fig. 7:**
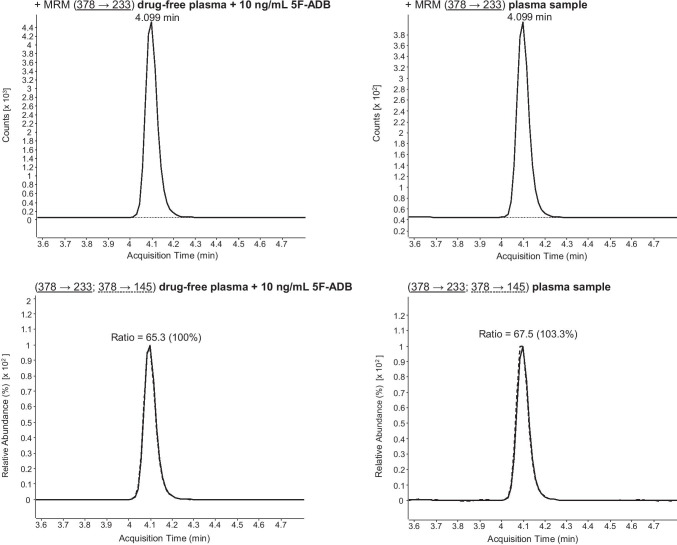
Confirmation of 5F-ADB (5F-MDMB-PINACA) via LC-ESI-MS/MS analysis (using the standard LC gradient) of plasma sample. MRM transitions and ratios of 5F-ADB (5F-MDMB-PINACA) of a drug-free plasma sample, spiked with 5F-ADB, and the plasma sample. For explanation: the respective sample can be found in the columns, the upper row shows the first MRM transition and the lower row the second MRM transition as well as the ion ratios

**Fig. 8:**
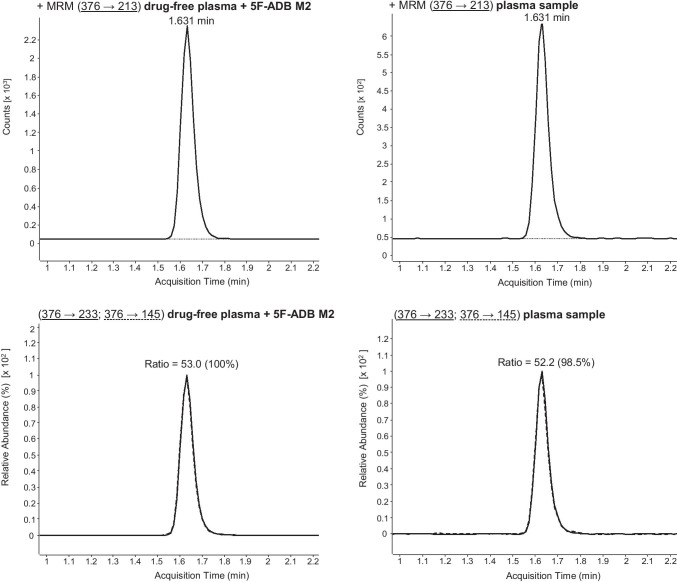
Confirmation of 5F-ADB *N*-(5-OH-pentyl) (“metabolite M2”, abbreviated with M2 here) via LC-ESI-MS/MS analysis (using the standard LC gradient) of plasma sample. MRM transitions and ratios of 5F-ADB M2 of a drug-free plasma sample, spiked with 5F-ADB M2, and the plasma sample. For explanation: the respective sample can be found in the columns, the upper row shows the first MRM transition and the lower row the second MRM transition as well as the ion ratios

The investigation was undertaken on a case of a 31-year-old man, who smoked *spice* for the first time. Routine STA and target screening for synthetic cannabinoids in the plasma sample, taken approximately 2 h after the incident, proved a recent use of cannabis and the synthetic cannabinoid 5F-ADB (5F-MDMB-PINACA) (Fig. 7). The plasma sample also revealed a positive finding for its metabolite 5F-ADB *N*-(5-OH-pentyl) (Fig. 8). In the *spice* sample, the synthetic cannabinoid 5F-MDMB-PICA could also be detected. However, this substance was not found in the plasma sample. The consumption of 5F-ADB (5F-MDMB-PINACA) might have led to the side effects the man suffered (panic attack, irritability, aggression, psychosis, tachycardia, heart and chest pain and circulatory collapse). It is questionable whether a co-use or a recent use of cannabis contributed to the severity of the side effects. However, this case once again underlines the health risks of synthetic cannabinoid use.

## Data Availability

Not applicable.
