# Correction to: Analytical findings in a non-fatal intoxication with the synthetic cannabinoid 5F-ADB (5F-MDMB-PINACA): a case report

**DOI:** 10.1007/s00414-022-02894-y

**Published:** 2022-10-21

**Authors:** Franziska Gaunitz, Hilke Andresen‑Streichert

**Affiliations:** Institute of Legal Medicine, University of Cologne, Faculty of Medicine and University Hospital, Cologne, Germany

**Correction to: International Journal of Legal Medicine (2022) 136:577–589**

**https://doi.org/10.1007/s00414-021-02717-6**

The online version contains an error in Fig. 3. Since the left part of Fig. 3 is missing in this publication, the authors would like to submit the missing figure herewith. This is now correctly displays the figure.

**Fig. 3:**
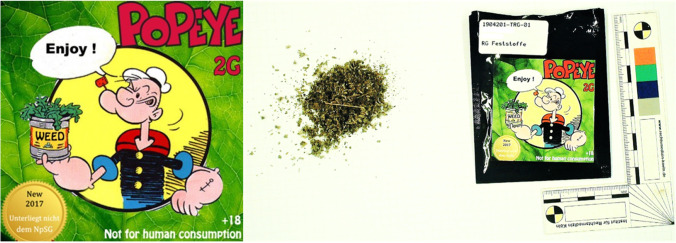
Label of “Popeye 2G, weed” (downloaded from https://lsd-blotter.com/legal-Smoke/Popeye-Legal-2g-R%C3%A4uchermischung, on December 18.^th^ 2020) (on the left) and *spice* sample of the here presented case (on the right)

